# High level of pattern glare in major depressive disorder

**DOI:** 10.1186/s12888-019-2399-6

**Published:** 2019-12-21

**Authors:** Xiongwei Qi, Huanhuan Fan, Xiao Yang, Yayun Chen, Wei Deng, Wanjun Guo, Qiang Wang, Eric Chen, Tao Li, Xiaohong Ma

**Affiliations:** 1 Psychiatric Laboratory and Mental Health Center, West China Hospital of Sichuan University, Chengdu, Sichuan 610064 People’s Republic of China; 2 West China Brain Research Center, West China Hospital of Sichuan University, Chengdu, China; 3 Department of Psychiatry, The University of Hong Kong, Queen Mary Hospital, Pokfulam, Hong Kong

**Keywords:** Major depressive disorder, Pattern glare, Visual deficits, Cortical hyper-excitability

## Abstract

****Background**:**

Visual deficits have been reported in abundance by recent studies on major depressive disorder. Pattern glare manifests as visual distortions, such as the symptoms of headache, glare, eyestrain, illusions of shapes, colors, and motion when viewing repetitive striped patterns, of which some can be observed in major depressive disorder. Inspired by what mentioned, the present study aims to explore whether there exists association between pattern glare and major depressive disorder and further attempts to explore possible clinical diagnostic value of pattern glare in major depressive disorder.

**Methods:**

Twenty-four patients diagnosed with major depressive disorder (MDDs group) were compared with 30 age-, gender- and education level-matched healthy control subjects (HCs group) on their visual stress with black-and-white gratings of different spatial frequencies-0.3 (low-SF), 2.3 (mid-SF), and 9.4 (high-SF) cycles per degree (c/deg)-which was named pattern glare test. The MDDs group divided into first episode medication-free group (fMDD) and recurrent medicated group (rMDD), comparisons of pattern glare scores (PGS) were performed within the MDDs group. We used Pearson and Spearman analysis to explore the relationship between some clinical indexes and pattern glare scores. ROC (receiver operating characteristic) curve was used to evaluate whether pattern glare test was able to discriminate patients and healthy controls.

**Results:**

The mid-SF pattern glare score significantly elevated in patients with major depressive disorder compared to control subjects. No differences of pattern glare scores were found between fMDD and rMDD. A significant negative correlation between mid-high difference and age in HCs group was found. There were no correlations between other variables and pattern glare scores. The mid-SF score has limited value in the diagnosis of major depressive disorder.

**Conclusions:**

We observed an increased level of pattern glare in patients with major depressive disorder, reflecting the existence of cortical hyper-excitability in major depressive disorder. The mid-SF score may have a value in understanding cortical excitability in major depressive disorder.

## Background

Major depressive disorder (MDD), affecting thousands of millions of people all around the world [1], is one of the most debilitating disease with the symptoms of low mood, declined interests and impaired cognition [2], along with somatic ones, such as headache [3–7] and insomnia [7]. Major depressive disorder was reported to be one of the five leading causes of years lived with disability (YLDs) in 2016 globally, resulting in 34.1 million of total YLDs [8].

Several lines of evidences show that there exist visual abnormities in patients with major depressive disorder. Photophobia, perceived dimness, anomalous preattentive processing of visual information and self-reported visual function loss were found to exists in patients with major depressive disorder [9–14]. Using the CogState battery (CSB) Chinese language version, a sensitive cognitive assessment instrument, an impairment in the visual, working, and verbal memory was found in first-episode, drug-free MDD patients in a Chinese population [15]. Longer visual search time was needed when detecting a target calling for difficult attentive search strategy in patients with MDDs than in controls [16]. Retinal contrast gain, visual contrast sensitivity, and visually evoked potentials (VEPs) amplitudes were found to decrease significantly in patients with MDD, by utilizing the method of pattern electroretinogram (PERG), subjective visual contrast test and VEPs recordings [17–20]. The normalization of decreased contrast gain after anti-depressant treatment was also reported, the therapies being varied markedly [21]. Developmentally and anatomically, the retina is thought to be an extension of the CNS and thus much of knowledge and discoveries obtained from eye research could be applied to the CNS [22].

Apart from the methods of PERG recording, VEPs recording and subjective visual contrast test, pattern glare test is another tool to explore vision abnormities. Some people can perceive visual perceptual distortions and discomfort, manifesting as the symptoms of eyestrain, headaches and glare, as well as illusions of shapes, colors, and motion, when viewing repetitive striped patterns [23, 24]. The extent of these experiences varies according to the character of the pattern and individual susceptibility [25]. High-contrast striped patterns, with spatial frequency around 3 cycles/degree and with equal width and spacing, tend to generate maximal effect [25]. The phenomenon depicted above has been named ‘pattern glare’ [26]. The term ‘Meares–Irlen syndrome’ or ‘visual stress’ was also used to describe the symptoms generated by pattern glare [27]. The neural mechanism underlying pattern glare is widely considered as cortical origin, that is, cortical hyper-excitability or poor cortical inhibition generated by deficiency of inhibitory mechanisms that are insufficient to contain the overexcited conditions [25, 27–33]. An increase of blood oxygenation in the visual cortex, evidenced by the fMRI BOLD signal, was found when subjects responded to square-wave gratings [34]. Jason J. Braithwaite and colleagues concluded that heightened levels of pattern glare can reflect increasement of cortical hyperexcitability associated with some possible abnormal experiences in some nonclinical populations [35]. Pattern glare has been assessed using The Wilkins and Evans Pattern Glare Test, which was first performed by Wilkins and Evans to identify individuals susceptible to visual stress by means of pattern related questionnaire, by counting scores for the number of visual illusions and discomfort induced by viewing three high-contrast gratings of spatial frequencies 0.5 cycles per degree (cpd), 3 cpd, and 12 cpd [27]. The application of pattern glare test in a great variety of neurological conditions, such as synaesthesia, autism, myalgic encephalomyelitis, multiple sclerosis, stroke, and reading ensued [36–43]. To the best of our knowledge, there is no paper reporting the association between major depressive disorder and pattern glare.

Inspired by the above-mentioned evidences that patients with major depressive disorder display various vision abnormities and pattern glare-related symptoms, such as headache and photophobia, we presume high level of pattern glare in the disease, which is assessed by using the pattern glare test. Meanwhile, the current way to diagnose major depressive disorder, depending mainly on the self-reporting of symptoms and mental evaluation, accompanies with the chance of misdiagnosis, which calls for a reliable biomarker with good accuracy for identifying the disease [44]. We attempted to explore possible diagnostic value of pattern glare test, as a potential biomarker, in major depressive disorder.

## Methods

### Participants

We recruited 24 patients diagnosed with major depressive disorder (MDDs group) and 30 age-, gender- and education level-matched healthy controls (HCs group) for the study; all the participants were right-handed. The study was conducted in accordance with the Declaration of Helsinki and was approved by the West China Hospital of Sichuan University ethics committee. All the 54 subjects provided written informed consent.

The 24 MDDs were recruited at the Mental Health Centre of West China Hospital, Sichuan University, People’s Republic of China. Inclusion criteria of MDDs group were the current diagnosis of a major depressive episode according to DSM-IV (Diagnostic and Statistical Manual of Mental Disorders, fourth edition) – Patient Version (SCID-P) criteria classified as either MDD with single episode or recurrent episode [45]. Exclusion criteria were as follows: [1] were younger than 18 years or older than 60 years, pregnant or breast feeding, or mentally retarded [2]; had major physical diseases, such as traumatic brain injury, encephalitis, epilepsy, or endocrine disease [3]; had a history of psychotic symptoms during the whole disease duration [4]; had other axis I disorders, such as generalized anxiety disorder, obsessive-compulsive disorder, or drug or alcohol abuse [5]; had other disease that can induce pattern glare, such as dyslexic, autistic, multiple sclerosis, migraine, stroke [6]; had any difficulties in completing the test, such as suffer from eye disease and impaired visual acuity.

The 30 healthy controls (HCs) were recruited locally by advertisement and were screened for a lifetime history or current depressive episode and other neuropsychiatric illness using the Structured Clinical Interview (SCID-NP) in the DSM-IV Non-Patient Edition [46]. We also excluded the controls whose first-degree relatives had any psychiatric illness or had a current or history of depression or other axis I disorders and assured that all the HCs had no other diseases which can induce pattern glare.

All participants were assessed immediately after recruitment in our study. All subjects were confirmed to be within the normal vision acuity utilizing the Snellen chart [47–49]. The severity of diseases was assessed using a 17-item Hamilton Depression Rating Scale (HAMD). Ten of the 24 depressive patients suffered from first episode and were all medication-free, while the other 14 ones from recurrent episodes and medicated.

### Pattern glare test

Pattern glare test has been regarded as a simple but established way to measure visual stress which reflects cortical hyperexcitability [25, 35]. The pattern glare test consists of three patterns with different spatial frequency, all of which were presented at 40 cm viewing distance and were performed in order from low spatial frequency to high. The first presentation, with low spatial frequency (low-SF) of 0.3 cycles per degree (cpd), is unlikely to induce any distortions or discomforts and is designed to ensure subjects provide true answers; The second pattern is presented with a mid-spatial frequency (mid-SF) gratings of 2.3 cpd and is able to induce maximum visual distortions in susceptible subjects; The third one with a higher spatial frequency (high-SF) of 9.4 cpd is expected to induce fewer symptoms than the second pattern. For every pattern, participants were instructed to fixated on the central of the presentation for 5 s, after which they were asked (yes or no) whether they experienced the following 15 symptoms: red, green, blue, yellow, bending of lines, blurring of lines, shimmering of lines, flickering, fading, shadowy shapes among the lines, pain, discomfort, nausea, dizziness, unease [50]. The numbers of the yes responses to the above symptoms were summed to generate a pattern glare score for each of the gratings. We also obtained an indirect score by subtracting the score of high-SF grating from the score of the mid-SF grating, namely mid-high difference variable.

### Statistical analysis

We used SPSS 25.0 software (IBM Corporation, Armonk, NY, USA) for Win10 to analyze all the data with a significance level of *p* = 0.05 used throughout the analysis. Continuous variables were presented as mean(M) and standard deviation (SD) and relative ratio was used to describe categorical variable. Comparisons of demographic characteristics and pattern glare scores between MDDs group and HCs were performed. The MDDs group was then divided into first episode medication-free group (fMDD) and recurrent medicated group (rMDD) to compare the pattern glare scores between them. Before the statistical analysis of the data, the quantitative variables were checked for normal distribution. Quantitative variables normally distributed were compared using two-sample t test, while those non-normally distributed were analyzed using Mann-Whitney U test. Categorical variable was compared using chi-square test. Spearman and Pearson analysis were utilized to detect whether there exist correlations between some indexes (i.e. age, HAMD score and illness duration) and pattern glare scores in both MDDs and HCs group. ROC (receiver operating characteristic) curve was used to evaluate whether pattern glare test was able to identify patients and healthy controls. Full details of data analyses were given in additional files.

## Results

### Demographic characteristics

Demographic data presented that MDDs group and HCs group were well matched for age (M ± SD; MDD: 30.50 ± 10.714; HCs: 27.70 ± 6.287; *P* = 0.265), gender distribution (male/female; MDDs: 9/15; HCs: 14/16; *P* = 0.498) and education years (M ± SD; MDD: 14.92 ± 2.412; HCs: 16.00 ± 2.421; *P* = 0.108). The 24 MDDs exhibited a HAMD score of 22.21 ± 4.334 (M ± SD), indicating that the patients were going through a depressive episode by the time of entering the study. The basic characteristics of the two groups were shown in Table 1.

**Table 1:** Demographic and clinical data of MDDs and HCs groups

Variables	MDDs	HCs	*P* value
Age (years)	30.50 ± 10.714	27.70 ± 6.287	0.265^*b*^
Gender (male/female)	9/15	14/16	0.498^*a*^
Education level (years)	14.92 ± 2.412	16.00 ± 2.421	0.108^*b*^
HAMD score	22.21 ± 4.334	–	–
Illness duration (months)	42.21 ± 56.628	–	–

*P*^*a*^ *=* Chi-square test; *P*^*b*^ = two-sample t test; HAMD = Hamilton Depression Rating Scale; Descriptive variables were described with mean ± standard deviation; Gender was described with relative ratio; *P* < 0.05 was considered as statistically significant.

### Differences of pattern glare scores between MDDs and HCs

The two groups differed significantly in pattern glare score of mid-SF grating (two-sample t test: *P* = 0.023), mid-SF score being significantly higher in MDDs group than the in HCs group. There were no statistical differences in pattern glare scores of low-SF grating (Mann-Whitney U test: *P* = 0.485), high-SF grating (Mann-Whitney U test: *P* = 0.092) and the mid-high difference (two-sample t test: *P* = 0.731) between the two groups (Table 2). The result indicated elevated level of pattern glare was induced by the mid-SF pattern rather than the other two gratings.

**Table 2:** Comparisons of PGS between MDDs and HCs

Variables	MDDs	HCs	*P* value
PGS of low-SF(p1)	1.00 ± 1.668	0.57 ± 0.935	0.485
PGS of mid-SF(p2)	4.58 ± 2.552	3.13 ± 1.776	0.023
PGS of high-SF(p3)	3.42 ± 2.669	2.20 ± 1.937	0.092
mid-high SF difference(s)	1.17 ± 1.949	0.97 ± 2.236	0.731

Data were described with mean ± standard deviation; *P* < 0.05 was deemed to be statistically significant.

### Comparisons of PGS between fMDD and rMDD

No statistical differences in pattern glare scores between the first episode medication-free group (fMDD) and recurrent medication undergoing group (rMDD) were found (Table 3).

**Table 3:** Comparisons of PGS between fMDD and rMDD

Variables	fMDD	rMDD	*P* value
PGS of low-SF(p1)	1.70 ± 2.214	0.50 ± 0.941	0.094
PGS of mid-SF(p2)	5.10 ± 2.283	4.21 ± 2.751	0.414
PGS of high-SF(p3)	3.30 ± 3.164	3.50 ± 2.378	0.861
mid-high SF difference(s)	1.80 ± 1.874	0.71 ± 1.939	0.201

fMDD = first episode medication-free patients with MDD; rMDD = recurrent medication-ongoing patients with MDD; Data were described with mean ± standard deviation; *P* < 0.05 was considered to be statistically significant.

### Correlations between pattern glare scores and some clinical indexes

Of all the correlation analysis, there was a significant negative correlation between mid-high difference and age in HCs group only (Spearman correlation analysis: *r* = − 0.421, *P* = 0.021); no any other significant correlations were found between pattern glare scores and all the other variables (Table 4).

**Table 4:** Correlations between pattern glare scores and several clinical indexes

	Group	Age	HAMD score	Illness duration
PGS of low-SF gratings (p1)	MDDs	*r* = − 0.181	*r* = − 0.163	*r* = − 0.049
		*p* = 0.397	*p* = 0.447	*p* = 0.821
	HCs	*r* = 0.008		
		*p* = 0.968		
PGS of mid-SF ratings (p2)	MDDs	*r* = − 0.277	*r* = 0.079	*r* = − 0.337
		*p* = 0.190	*p* = 0.714	*p* = 0.107
	HCs	*r* = − 0.263		
		*p* = 0.161		
PGS of high-SF gratings(p3)	MDDs	*r* = −0.154	*r* = 0.126	*r* = − 0.148
		*p* = 0.474	*p* = 0.556	*p* = 0.491
	HCs	*r* = 0.164		
		*p* = 0.388		
Scores of mid-high SF(s)	MDDs	*r* = −0.184	*r* = 0.032	*r* = − 0.252
		*p* = 0.390	*p* = 0.883	*p* = 0.235
	HCs	*r* = −0.421		
		*p* = 0.021		

*P* < 0.05 was considered to be statistically significant.

### Clinical diagnostic value of mid-SF pattern glare score in major depressive disorder

**Fig. 1:**
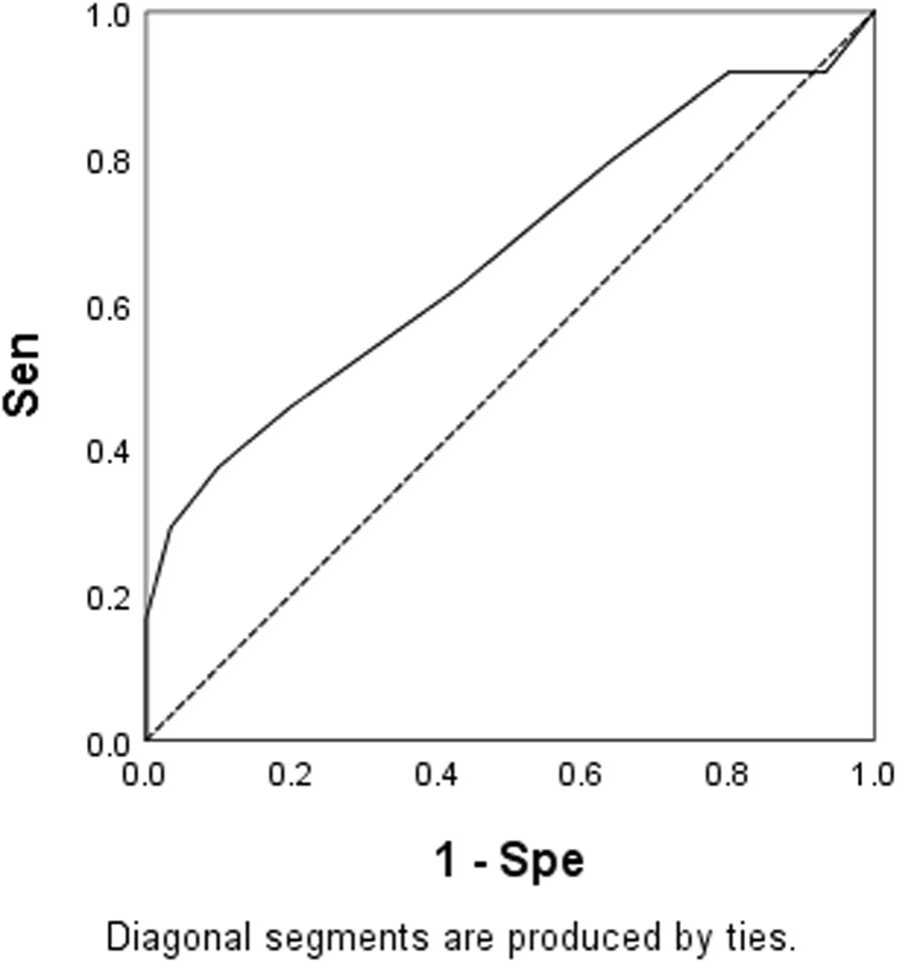
ROC curve of ‘p2’. AUC: 0.668 (95% CI:0.519–0.817), *p* = 0.035

Since the mid-SF pattern glare score exhibited significant difference between MDDs group and HCs group, we conducted ROC analysis and found the area under the curve of mid-SF scores was 0.668 (*p* = 0.035, Fig. 1), indicating the mid-SF has limited value to discriminate MDDs from HCs.

## Discussion

We demonstrated that there exists high level of pattern glare in major depressive disorder. The MDDs group scored higher in the mid and high frequencies but not low frequency pattern gratings than the HCs group. Previous studies have found that specifically the mid-SF pattern that would induce most distortions. The mid-SF score and mid-high difference are the two most distinctive indications of pattern glare [23, 36, 43]. Significantly negative correlation between mid-high difference and age in HCs group was found. Our performance of ROC analysis using pattern glare score of mid-SF showed the score possesses limited value of identifying the depressed and the healthy.

Several lines of evidences reported decreased GABA levels in occipital cortex, anterior cingulate cortex (a prefrontal cortical region) and the dorsomedial/dorsal anterolateral prefrontal ROI in MDD subjects and even in recovered depressed patients [51–55].

The association between altered GABA levels and vision abnormity has also been reported frequently. Visual deficit-poor vision-was found to be rendered by loss of dendritic cell factor 1 through the GABA system in mouse primary visual cortex [56]. In the early stage of type 2 diabetes, occipital cortical GABA has been reported to be a novel predictor of visual psychophysical performance, that is, speed and achromatic discrimination thresholds [57]. The application of GABA and its agonist to senescent macaques lead to improved orientation and direction, an enhanced ability to signal visual stimuli, combined with decreased visual responsiveness and spontaneous activity [58]. Increased visual cortical GABA levels was found to be correlated with longer percept durations [59]. Remarkably reduced occipital GABA concentrations has been reported to be possibly responsible for the visual problem in first-episode, unmedicated MDD [60].

These findings thrown some light on interpreting pattern glare via the GABA system. The symptoms of pattern glare are more obvious under binocular than monocular conditions, GABA being the neurotransmitter whose interneurons are responsible for the combination of the input from the two eyes in the cortex, which suggest a role of GABA in the mechanism of pattern glare [24, 27, 61]. Additionally, the decreased GABA levels in MDD lead to deficient cortical inhibition or cortical hyper-excitability and thus high level of pattern glare, cortical hyper-excitability being deemed to be the mechanism of pattern glare.

Whether psychiatric stress or other psychiatric conditions, such as anxiety or schizophrenia, plays a role in increasing pattern glare score could be concerned. The patients in our study were free of other psychiatric symptoms. It is well worth involving more psychiatric diseases, such as anxiety disorder, schizophrenia and obsessive-compulsive disorder, in the future study of pattern glare.

The result of significantly negative correlation between mid-high SF difference and age in HCs group showed an overall decreasing level of pattern glare with age, which is consistent with previous finding that there existed a significant inverse correlation between age and the pattern glare score for the 3 cpd. The result indicated increased cortical inhibition with age, agreeing with a recent finding that visual cortical GABA levels increased in older adults [59]. Primary cortex synchrony, contributing to cortical hyperexcitability, was reported to get losing with age in monkey, which leads to a decreasing level of pattern glare with age [62, 63].

Many optical changes in the eye with age including lens changes, increased aberrations and senile pupillary miosis can result in a reduction of the contrast sensitivity function; these changes may give rise to lower level of light reaching the retina and might have an influence on the results of the pattern glare test with age [27, 64]. The loss of sensory acuity with age may also be responsible for the decreased pattern glare scores [27]. However, the MDDs group displayed no negative correlation between age and pattern glare scores. Since the age between the MDDs and the HCs did not differ significantly, this result indicated relatively reduced cortical inhibition in MDDs group compared to the HCs group, which is consistent to our assumption.

The ROC analysis of pattern 2 displayed limited clinical diagnostic value of identifying the depressed and the healthy, the area under the curve being not so large. But we hold that the test deserves further exploration, for it is rather easy to implement, but may represent a new potential biomarker of diagnosing MDD. Replication of the test with large sample size is warranted to explore whether it is possible to find the diagnostic value of pattern 2 more powerful.

Our study has two limitations. First, though there were no significant differences in pattern glare score between first episode mediation-free subjects and recurrent medication-ongoing patients, it is unclear whether the effect of disease episode numbers on pattern glare scores and the effect of medicine can influence each other. Three groups, including healthy controls, first-episode medication-free subjects and the subjects after medication therapy can be set to explore the effects of medicine on pattern glare score in future study. Second, our study was limited by a small sample size. The results need to be verified in future study for it is the first time the pattern glare test has been applied to major depressive disorder.

## Conclusions

In conclusion, we feel that our study is valuable, for it is a rather simple test with a promise of being a diagnostic tool of major depressive disorder. We mainly found the score difference in mid-SF of 2.3 cpd gratings between MDDs and HCs group was significant and the pattern 2 has limited value in the diagnosis of major depressive disorder. Since the study is the first one applying the pattern glare test in MDD, well-designed replication is needed to further verify the outcome.

## Data Availability

The datasets used and analyzed during the current study are not publicly available due to no permission of the ethics committee, but are available from the corresponding author on reasonable request.

## Abbreviations

HAMD: Hamilton Depression Rating Scale
HC: Healthy control
MDD: Major depressive disorder
PGS: Pattern glare score
ROC: Receiver operating characteristic
